# Decomposing Cost Efficiency in Regional Long-term Care Provision in Japan

**DOI:** 10.5539/gjhs.v8n3p89

**Published:** 2015-07-13

**Authors:** Yasuhiro Yamauchi

**Affiliations:** 1 Faculty of Economics, Tezukayama University, Nara, Japan

**Keywords:** cost efficiency, data envelopment analysis, non-parametric frontier approach, long-term care, Japan

## Abstract

Many developed countries face a growing need for long-term care provision because of population ageing. Japan is one such example, given its population's longevity and low birth rate. In this study, we examine the efficiency of Japan's regional long-term care system in FY2010 by performing a data envelopment analysis, a non-parametric frontier approach, on prefectural data and separating cost efficiency into technical, allocative, and price efficiencies under different average unit costs across regions. In doing so, we elucidate the structure of cost inefficiency by incorporating a method for restricting weight flexibility to avoid unrealistic concerns arising from zero optimal weight. The results indicate that technical inefficiency accounts for the highest share of losses, followed by price inefficiency and allocation inefficiency. Moreover, the majority of technical inefficiency losses stem from labor costs, particularly those for professional caregivers providing institutional services. We show that the largest share of allocative inefficiency losses can also be traced to labor costs for professional caregivers providing institutional services, while the labor provision of in-home care services shows an efficiency gain. However, although none of the prefectures gains efficiency by increasing the number of professional caregivers for institutional services, quite a few prefectures would gain allocative efficiency by increasing capital inputs for institutional services. These results indicate that preferred policies for promoting efficiency might vary from region to region, and thus, policy implications should be drawn with care.

## 1. Introduction

Many developed countries face a growing need for long-term care provision because of population ageing. Japan is one such example, given its population's longevity and low birth rate. As Aboagye et al. (2014) contend, social security to the elderly may be improved and extended by ensuring that the traditional in-kind social security system arranged by the extended family go hand-in-hand with the formal social security structure. Considering this, in 2000, the Japanese government initiated a mandatory social long-term care insurance (LTCI) system, which is operated by both the central and the local governments (Ikegami, 1997; Campbell & Ikegami, 2000). Based on the physical and mental status of the individual, irrespective of his/her income or family situation, this system makes long-term care a universal entitlement for every Japanese citizen aged 65 years and above. LTCI basically operates on social insurance principles: recipients receive services and choose providers, but cash allowances are not provided. Out-of-pocket expenses for elderly beneficiaries amount to 10% of the expenses for services received. The remaining revenues from premiums, national taxes, prefectures, and municipalities are 50%, 25%, 12.5%, and 12.5%, respectively.

As this system has proven to be popular among the general public, who have widely accepted it as a normal feature of social policy, demand for LTCI services has significantly increased, giving rise to the problem of runaway expenditures. Over the program's 12 years of operation since FY 2000, in which LTCI was established, the number of persons certified by all Japanese local governments to be in need of long-term care increased by 144%, to 5.33 million, far exceeding the growth (38%) of the population aged 65 years and above during the same time. Public expenditure for long-term care in FY 2012 amounted to 8.9 trillion yen (= 98 billion U.S. dollars), 1.88% of Japan's nominal GDP, which is higher than median value of OECD countries (OECD, 2013).

Furthermore, an ageing population, declining birth rates, and prolonged economic problems have forced Japan's policymakers to consider containing the costs of providing long-term care. Over the next decade, the proportion of Japan's elderly people who are at least 75 years old and prone to needing long-term care is expected to increase by 50%, to approximately 22 million. In recent years, the Japanese government implemented major LTCI reforms to restrict costs by assigning room fees and board expenses for institutional care, proactively promoting in-home services, and increasing emphasis on preventive services for those with lower needs and those at risk of needing future care.

This study examines the efficiency of Japan's regional LTCI services. We perform a data envelopment analysis (DEA) on 47 regions by prefecture, Japan's subnational jurisdiction. Numerous recent studies have measured the efficiency of long-term care using DEA, a linear programming technique developed by Charnes et al. (1978) based on earlier works (Farrell, 1957) and related methods. According to Hollingsworth (2008), who presents a review of published papers on frontier efficiency measurement in healthcare, after hospitals, nursing homes constitute the second-most common application for this method.

In terms of efficiency of long-term care, previously studied subjects include nursing homes, group homes, visiting nurse service agencies, municipalities, long-term care wards, geographically defined community care access centers, and similar institutions. The most common subject for study is nursing homes. Studies on nursing homes in the U.S., the Netherlands, and Italy include those by Sexton et al. (1989) and Nyman et al. (1990); Kooreman (1994); and Garavaglia et al. (2011), respectively. Although many studies have investigated efficiency in long-term care provision, there are relatively few studies on a regional long-term care system managed by a local public entity, which is the focus of this study. The latter include literature on municipalities (e.g., Erlandsen & Førsund, 2002; Hougaard et al., 2004; Laine et al., 2005; Borge et al., 2009). To elucidate the structure of cost inefficiency, we apply the formula used by Thanassoulis et al. (2012), which is a modification of the method suggested by Tone (2002) and Tone and Tsutsui (2007), and we deconstruct cost efficiency into technical, allocative, and price efficiencies. This study is unique in that it is the first to decompose cost efficiency into various efficiencies including price efficiency in the regional long-term care context.

This study is structured as follows. Section 2 provides the methodology and data used for estimating the efficiency of long-term care. Section 3 presents the estimation results and is followed by the discussion and concluding remarks in Section 4.

## 2. Methods and Data

### 2.1 Methods

We perform a DEA using the data of Japan's 47 prefectures. DEA is a non-parametric frontier approach based on linear programming that converts multiple input and output measures of a decision-making unit (DMU) into a single comprehensive measure of its productive efficiency by comparing related DMUs (Charnes et al., 1978). An advantage of DEA is that it does not assume frontiers to possess a particular functional form, unlike in the estimation of stochastic frontier models (Crivelli et al., 2002). DEA also differs from ordinary least squares estimation, which is based on comparisons relative to an average unit. We measure the monetary values of inefficiency, which are then separated into several components by input factor (Thanassoulis et al., 2012).

This paper considers *n* DMUs and *m* inputs for producing *s* outputs. We denote the input and output vectors as and x*j*∈*R^m^* and y*j*∈*R^s^*, respectively, for each DMU*_j_* (*j* =1,…,*n*). We define the input and output matrices as and X=(x1,…,x*n*)∈^*Rm*×*n*^ and Y=(y1,…,y*n*)∈ *R*^*s*×*n*^, respectively. We assume that X>0 and Y>0. For each DMU*_j_* (*j* =1,…,*n*), we denote the input factor price vector for input x*_j_* by w*_j_*∈ *R^m^* and the input factor price matrix as W=(w1,…w*_n_*)∈*R^m^×*n**. For DMU*_j_*, the actual total input cost *C_j_* is calculated as follows:


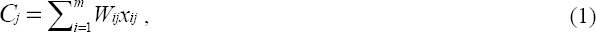


where *x_ij_* is the amount of the *i*th input used by DMU*_j_*, and *w_ij_* is the input factor price. We assume that elements *w_ij_x_ij_*,…,*w_mj_x_mj_*, are denominated in homogenous units, that is, in Japanese yen, such that the summation is measurable.

First, we calculate the technical efficiency of Japan's long-term care provision. The production possibility set *P* is defined as


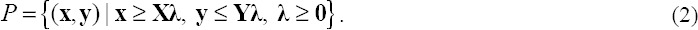


The technical efficiency of θ* DMU*_j_* is measured using the input-oriented variable returns to scale (VRS) model (Banker et al., 1984):


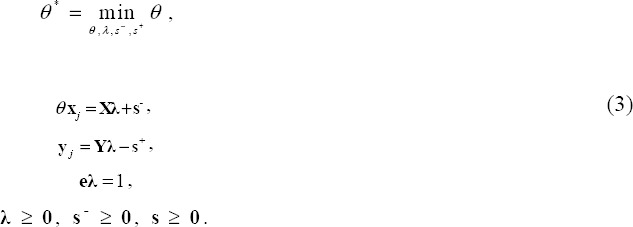


subject to


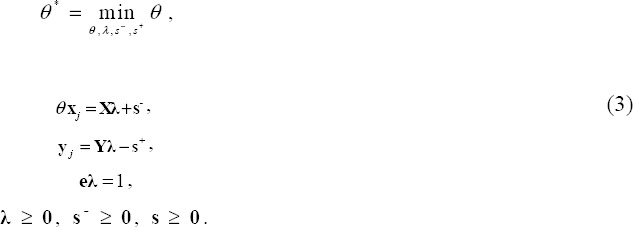


Since we use cross-sectional data of 47 regions with uncontrollable heterogeneity in population size, we employ the VRS model. In addition, we incorporate a method for restricting weight flexibility to avoid unrealistic concerns arising from zero optimal weight. The method by Wong and Beasley (1990) for restricting weight flexibility uses the following proportions:


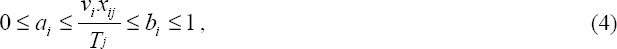


where *a_i_* and *b_i_* are regarded as suitable lower and upper limits, respectively; 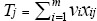 is the total input; and indicates the weight to be attached to the input measure.

The specification of the limit [*a_i_*,*b_i_*] is a value judgment (Wong & Beasley, 1990). However, we have no consensus on the relative importance of each input for Japan's long-term care provision. Hence, we specify the limit as follows:


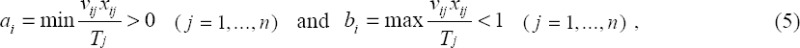


where *v_ij_* is estimated by the basic VRS model without restricting its weight flexibility.

Let (θ*, λ*, s^-^*, s^+^*) be the optimal solution for model (3) after adding weight restrictions. The projection of the efficiency frontier can then be given by


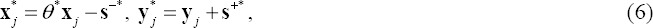


Where 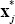 indicates the vector of the technically efficient inputs for DMU*_j_* for producing 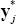.

The corresponding technically efficient total input cost for DMU*_j_* can be expressed as follows:


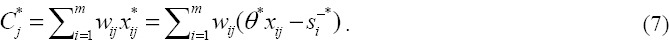


Loss due to this technical inefficiency is calculated as follows:


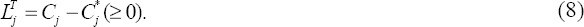


The technical efficiency TE of DMU*_j_* is defined as follows:


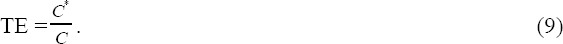


Next, we calculate the cost efficiency of Japan's long-term care provision. Traditional cost efficiency is defined as follows:


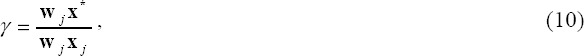


where x* is the vector obtained as the optimal solution to the following linear program:


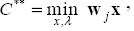


subject to


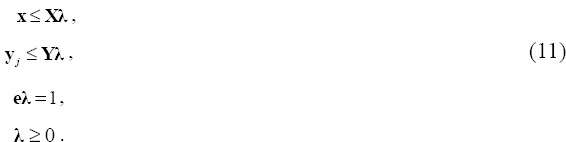


The optimal solution from this model yields the minimum cost C** at which DMU*_j_* could secure its outputs, given the unit costs.

Losses due to this cost inefficiency are calculated as follows:


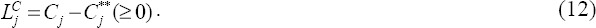


As cost efficiency is the product of technical and allocative efficiencies, the allocative efficiency AE of DMU*_j_* is defined as follows:


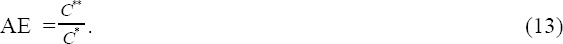


AE reflects the adjustment to the optimal input mixture based on the given input price ratio. Loss due to this allocative inefficiency is calculated as follows:


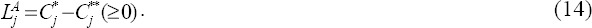


Next, we calculate the price efficiency of Japan's long-term care provision. Considering the input price differences caused by unit price variations between DMUs, costs can be reduced by altering input factor prices (Tone & Tsutsui, 2007). We use Thanassoulis et al.'s (2012) approach, wherein price efficiency is estimated using the traditional cost model, which identifies the input volumes that will minimize costs given a DMU's price. Then, using the optimal input volumes from the traditional cost model (*x_ij_*), we compute the corresponding cost vectors for each DMU*_j_*: X̄=(x̄_1_,...x̄_n_) with x̄_*j*_ =(w1_*j*_ *x*1**_j_,…,*w_mj_*x_mj_**)*^T^*. Next, we use these cost vectors in the model as follows:


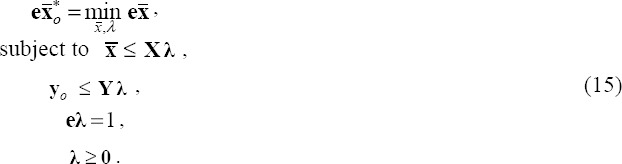


This model seeks to minimize a DMU's aggregate costs, controlling for output levels. It identifies cost savings by altering unit prices and input mix simultaneously (Thanassoulis et al., 2014).

Let the optimal (minimum) cost estimated for DMU*_j_* by the price efficiency model be denoted as C***.

Loss due to this price inefficiency is calculated as follows:


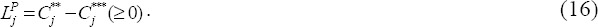


As the overall cost efficiency including price efficiency (PE) is C***/*C_j_*=(C**/*C_j_*)×(C***/C**), the PE of DMU*_j_* is defined as follows:


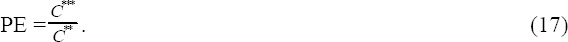


In accordance with Thanassoulis et al.'s (2012) method, our methodology assumes that volumes change first, and prices change residually.

### 2.2 Data

We use data of 47 regions by prefecture. This level of analysis was chosen because although municipalities serve as LTCI insurers and administer LTCI based on the national government's guidelines, prefectures provide municipalities with technical and administrative support and have the authority to decide on the number of LTC providers. In addition, as pointed out by Kawase and Nakazawa (2009), there can be significant elderly migration at the municipality level depending on the availability of institutional services. The current study's sample size is limited to, but comparable with, those used in other DEA studies on long-term care. The data are obtained from Japan's Ministry of Health, Labour and Welfare (MHLW) and other relevant entities and cover FY2010. The datasets are obtained from several sources. Input data are obtained from the Survey of Institutions and Establishments for Long-term Care (MHLW). Capital cost data are gathered from the Briefing Survey on Economic Conditions in Long-term Care (MHLW). The Survey on Employment in Long-term Care (Care Work Foundation, 2011) provides the labor unit cost data, and the Report on Condition of Long-term Care Projects (MHLW) provides the output data. Although DEA has a limitation in terms of measurement errors, they are considered to be small as the survey forms were completed by public officers or administrative staff of long-term care providers, all of whom are assumed to be familiar with the actual situation. These data include the most recent LTCI reports before the Great East Japan Earthquake.

The model comprises six inputs with unit costs and one united output. The number of variables is chosen to balance the trade-off between the model's descriptive and discriminatory powers. Capital input variables include the following: (a) the admission capacity of institutional services and (b) number of providers for in-home services. Labor input variables are numbers of (c) professional caregivers for institutional services, (d) professional caregivers for in-home services, (e) medical nurses, and (f) other staff (allied health professionals and office workers). The admission capacity of institutional services excludes that of day services, the number of providers for in-home services and professional caregivers for institutional services contains those for day services, and the number of professional caregivers for in-home services excludes those for day services. The differences in the above-mentioned inclusions/exclusions depend on the definitions and ranges of data in input factor prices (unit costs). Medical nurses include regular nurses, assistant nurses, healthcare nurses, and maternity nurses. “Other staff” refers to care managers, pharmacists, nutritionists, physiotherapists, occupational therapists, speech–language–hearing therapists, psychiatric social workers, care counselors, life counselors, cooks, dental hygienists, and welfare equipment counselors. Labor input variables [(c)–(f)] are calculated to yield their full-time equivalents.

Input factor prices (unit costs), using input variables for capital costs, are weighted average depreciation and interest on borrowing for (g) institutional services (per bed) and (h) in-home services (per office). The availability of factor price data for capital costs is extremely limited. As a result, we necessarily use unit cost data categorized by five levels of urbanization. Meanwhile, labor costs include per-person scheduled salaries and wages of (i) professional caregivers for institutional services, (j) professional caregivers for in-home services, (d) medical nurses, and (e) other staff.

For outputs, we calculate the weighted number of persons requiring care based on the certification of long-term care as (m) requirement of care. This factor is selected for the following reasons. A problem with analyzing the efficiency of providing care is the difficulty in measuring its conceptual output, namely, improved health status, or more generally, improved quality of life (Kooreman, 1994). DEA and related efficiency analyses frequently employ the concept of case mix—the type of disorder and its severity in patients treated by a unit; however, to the best of our knowledge, Japan's government does not collect outcome data or process performance systematically. This limits the possibility of employing outcome-related measures for determining quality of care. Therefore, our choice for this variable depends on the particulars of Japan's long-term care system. The provision of some services requires a Care-Needs Certification in the Long-Term Care Insurance System. Under Japan's Long-Term Care Insurance Act, “An insured person that intends to receive long-term care benefit shall obtain certification by a municipality pertaining to the fact that the said insured person qualifies as a person requiring long-term care and as to the category of condition of need for long-term care for which said insured person qualifies.” Therefore, this analysis employs the weighted number of persons certified as requiring care for the annual workload weighted by the degree of the seriousness of the disorder. We use “estimated total care minutes per day” (ETCM) as the weighting index for disorder seriousness (Table 1). ETCM is the official criterion for Japan's LTC Insurance Care Needs Levels and shows the estimated times needed for daily long-term care (Tsutsui & Muramatsu, 2005). For example, for 2012, Tokyo recorded the following number of people dependent on healthcare: 61,205 needed “support 1” care; 55,969 needed “support 2” care; 75,410 needed “level 1” care; 79,411 needed “level 2” care; 60,833 needed “level 3” care; 56,732 needed “level 4” care; and 54,926 needed “level 5” care. Accordingly, we can show the index for requirement of care = 25 × 61,205 + 32 × 55,969 + 32 × 75,410 + 50 × 79,411 + 70 × 60,833 + 90 × 56,732 + 110 × 54,926 = 25,110,853.

**Table 1:** Criteria for LTC insurance care needs levels

Level of care needed	Estimated Total Care Minutes per Day
Not eligible	< 25
Support 1	25≤
Support 2 and Level 1	32≤
Level 2	50≤
Level 3	70≤
Level 4	90≤
Level 5	110≤

* As of 2010. ** We calculated the number of persons requiring care using “Estimated Total Care Minutes per Day” as the weighting index for seriousness.

The summary statistics for the input and output values are described in Table 2. The maximum values of input and output exceed the minimum numbers of input and output by more than 10 times. The maximum values of input and output belong to either Tokyo Metropolis [(a), (c), (e), (f), and (m)] or Osaka Prefecture [(b) and (d)], both of which have high populations. The minimum values belong to either Tottori Prefecture [(b), (d), (e), (f), and (m)] or Yamanashi Prefecture [(a) and (c)], both of which have low populations. We use DEA-solver Professional Version 9.0 to calculate the efficiency estimates (Cooper et al., 2007).

**Table 2:** Major dataset statistics

n = 47	Average	S.D.	Max	Min
Input data				
Capital				
(a) Admission capacity of institutional services	20,478	16,416	79,129	6,417
(b) Offices for in-home services	2,532	1,799	8,392	763
Labor				
(c) Professional caregivers for institutional services	15,577	11,042	50,528	4,991
(d) Professional caregivers for in-home services	3,584	3,638	18,286	745
(e) Medical nurses	3,614	2,402	11,667	1,199
(f) Other staff	8,581	6,073	29,724	2,725
Input factor prices (thousand yen*)				
Depreciation cost and interest on borrowing				
(g) Institutional services (per bed)	424	14.5	451	400
(h) In-home services (per office)	3,009	568	4,148	2,267
Scheduled salaries and wages (per capita)				
(i) Professional caregivers for institutional services	2,323	163.9	2,729	2,015
(j) Professional caregivers for in-home services	2,257	209.9	2,763	1,783
(k) Medical nurses	3,065	331.2	3,967	2,480
(l) Other staff	2,870	207.0	3,392	2,499
Output data						
(m) Requirement of care	60,24,084	48,71,280	2,51,10,853	17,48,422

Sources: The datasets for this study are obtained from (1) the Survey of Institutions and Establishments for Long-term Care (MHLW) for (a) to (f), (2) the Briefing Survey on Economic Conditions in Long-term Care (MHLW) for (g) to (h), (3) the Survey on Employment in Long-term Care (Care Work Foundation, 2011) for (i) to (l), and (4) the Report on Condition of Long-term Care Projects (MHLW) for (m).

* One thousand yen was equivalent to about 11 U.S. dollars in 2010.

## 3. Empirical Results

Table 3 presents a summary of estimated capital and labor costs (**w***_j_***x***_j_*) from our data for each prefecture. We note that these calculated costs differ from actual costs and do not contain operating costs except capital and labor costs. According to our data for 2010, the total estimated capital and labor costs for all prefectures were approximately 4.6 trillion yen (= 51 billion U.S. dollars). This table demonstrates that labor costs are significantly greater than capital costs. The labor costs of professional caregivers for institutional services (average: 37.9%) comprise the largest proportion of each area's long-term care costs. On the other hand, the labor costs of professional caregivers for in-home services (average: 7.6%) comprise the smallest proportion of each area's long-term care costs. This might mean that there is more potential to contain long-term care costs by promoting the labor productivity of professional caregivers in institutional services.

**Table 3:** Estimated costs according to the raw data

n = 47	Average	S.D.	Max	Min
Capital cost (million yen*)				
Institutional services	8,782	7,258	34,557	2,674
(%)	8.8%	0.7%	10.6%	7.1%
In-home services	7,616	5,302	22,546	2,078
(%)	8.1%	1.7%	12.6%	4.9%
Labor cost (million yen*)				
Professional caregivers for institutional services	36,971	28,564	1,37,886	12,632
(%)	37.9%	2.1%	42.3%	34.2%
Professional caregivers for in-home services	8,268	8,888	43,655	1,641
(%)	7.6%	1.9%	14.2%	4.8%
Medical nurses	11,525	8,991	46,281	3,575
(%)	11.8%	1.1%	14.1%	9.5%
Other staff	25,259	19,751	1,00,817	8,011
(%)	25.9%	1.3%	28.4%	23.1%

* One million yen was equivalent to about 11 thousand U.S. dollars in 2010.

Table 4 summarizes the results of technical efficiency (*TE*), allocative efficiency (*AE*), price efficiency (*PE*), and overall efficiency (*OE*). *OE* was calculated as follows:


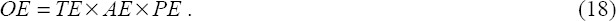


The average *OE* index is 0.794, with a minimum of 0.695. The average *TE* index is 0.914, which is lower than the average *AE* index (0.938) and the average *PE* index (0.929).

**Table 4:** Summary of estimated efficiency indexes

n = 47	Average	S.D.	Max	Min
Technical efficiency (TE)	0.914	0.067	1.000	0.786
Allocative efficiency (AE)	0.938	0.047	1.000	0.814
Price efficiency (PE)	0.929	0.049	1.000	0.794

Overall efficiency (OE)	0.794	0.065	1.000	0.695

Table 5 presents overall inefficiency losses and the factor-oriented decomposition of each estimated inefficiency loss. Inefficiency losses are derived by totaling the differences between the costs given by the raw data and the estimated optimum costs for each prefecture. As seen in the table, overall inefficiency losses amount to 922 billion yen (approximately 9 billion U.S. dollars), their ratio to the total cost being 19.9%. This result indicates substantial variations in efficiency across prefectures and reveals that the national-level efficiency potential is approximately 20%. Overall inefficiency losses by labor cost amount to 786 billion yen (85% of overall inefficiency losses), which is greater than overall inefficiency losses by capital costs (136 billion yen, which comprise 15% of overall inefficiency losses). This result shows that promoting labor productivity, rather than capital productivity, has more potential to contain long-term care costs.

**Table 5:** Overall inefficiency losses and factor-oriented decomposition

Inefficiency	Overall		Technical		Allocative		Price	
Capital cost (million yen*)								
Institutional services	45,313	4.9%	32,510	7.8%	4,667	1.9%	8,137	3.2%
In-home services	90,195	9.8%	43,692	10.5%	30,710	12.2%	15,793	6.2%
Labor cost (million yen*)								
Professional caregivers for institutional services	3,85,016	41.8%	1,65,274	39.5%	1,30,322	51.9%	89,420	35.4%
Professional caregivers for in-home services	36,553	4.0%	20,439	4.9%	-4,694	-1.9%	20,808	8.2%
Nurses	1,18,017	12.8%	50,739	12.1%	11,316	4.5%	55,962	22.1%
Other staff	2,46,809	26.8%	1,05,288	25.2%	78,871	31.4%	62,651	24.8%

Total loss (million yen*)	9,21,904	100.0%	4,17,942	100.0%	2,51,192	100.0%	2,52,770	100.0%
(Ratio of overall inefficiency loss)				(45.3%)		(27.2%)		(27.4%)
(Ratio of total cost)		(19.9%)		(9.0%)		(5.4%)		(5.5%)

* One million yen was equivalent to about 11 thousand U.S. dollars in 2010.

**Table 6:** Various cases of factor-oriented allocative inefficiency loss

	Case 1	Case 2	Case 3	Case 4	Case 5	Case 6	Case 7	Case 8	Case 9
Capital cost									
Institutional services	L	G	G	L	G	L	L	G	L
In-home services	L	L	L	G	L	L	L	G	G
Labor cost									
Professional caregivers for institutional services	L	L	L	L	L	L	L	L	L
Professional caregivers for in-home services	G	L	G	G	G	L	G	G	G
Nurses	L	G	L	L	G	G	G	L	G
Other staff	L	L	L	L	L	L	L	G	G

Number of prefectures	20	11	5	3	2	1	1	1	1

*Two prefectures have no allocative inefficiency loss. *The Ls indicate allocative inefficiency losses. The Gs indicate allocative efficiency gains.

Technical inefficiency losses amount to 418 billion yen (9.0% of estimated total costs), which is greater than both allocative inefficiency losses (251 billion yen; 5.4% of estimated total costs) and price inefficiency losses (253 billion yen; 5.5% of estimated total costs). These results show that approximately half (45.3%) of overall inefficiency losses stem from technical inefficiency losses. Most technical inefficiency losses can be traced to labor costs for professional caregivers in institutional services (39.5% of technical inefficiency losses).

Allocative inefficiency losses amount to 251 billion yen (5.4% of estimated total costs), and the majority of allocative inefficiency losses can be traced to labor costs for professional caregivers in institutional services (51.9% of net allocative inefficiency losses). Furthermore, we find that few allocative inefficiency losses arise from labor costs associated with professional caregivers providing in-home care services. Instead, providing in-home care services shows an efficiency gain. Table 6 shows the various cases (patterns) of factor-oriented allocative inefficiency losses. The Ls indicate the existence of allocative inefficiency losses. The Gs show the presence of allocative efficiency gains. For example, in Case 1, 20 prefectures would gain allocative efficiency by increasing labor inputs for professional caregivers for in-home services and decreasing other inputs. In Case 3, 5 prefectures would gain allocative efficiency by increasing capital inputs and labor inputs for professional caregivers for in-home services and decreasing other inputs. These results support government policy, which proactively promotes in-home services to make long-term care provision more efficient. But, although there is no prefecture that gains allocative efficiency by increasing the number of professional caregivers in institutional services, 18 prefectures would gain allocative efficiency by increasing capital inputs for institutional services. For example, in Case 2, 11 prefectures would gain allocative efficiency by increasing capital inputs for institutional services and labor inputs for nurses and decreasing other inputs. As illustrated in this example, preferred policies for promoting allocation efficiency vary from region to region.

Price inefficiency losses amount to 253 billion yen (5.5% of estimated total costs). When we decompose inefficiency losses by factor, we see that losses from labor costs for professional caregivers in institutional services exceed those from other factors. From this estimated result, we can conclude that lowering the labor rate of institutional services will significantly improve efficiency. However, such a strategy might incur the difficulty of securing sufficient employee numbers.

## 4. Discussion and Conclusion

In this study, we examined the efficiency of Japan's regional long-term care for 2010. We performed a DEA on 47 regions by prefecture, Japan's subnational jurisdiction. To elucidate the structure of cost inefficiencies in Japan's provision of long-term care, we applied the formula used by Thanassoulis et al. (2012), which is a modification of the methods proposed by Tone (2002) and Tone and Tsutsui (2007), for separating cost efficiency into technical, allocative, and price efficiencies.

The results of this study are as follows: (a) technical inefficiency accounts for the highest share of losses, followed by price inefficiency and allocation inefficiency; (b) the majority of technical inefficiency losses stem from labor costs, particularly those for professional caregivers providing institutional services; (c) the largest share of allocative inefficiency losses can also be traced to labor costs for professional caregivers providing institutional services; instead, the labor provision of in-home care services shows an efficiency gain; (d) however, a number of prefectures would gain allocative efficiency by increasing capital inputs for institutional services.

These results reveal substantial efficiency variations across prefectures and show national-level efficiency potential to be approximately 20%. This finding suggests that regional long-term care efficiency could be improved through better services and resource provision management. Thus, the first policy implication drawn from this study concerns reforming institutional service provision, which could help improve the efficiency of Japan's long-term care system. Despite structural changes to the LTCI system in 2000, the new program had minimal impact on institutional care providers (Ikegami et al., 2003). One could argue that institutional services show scope for improvement through higher labor productivity, especially via technical efficiency. As Japan's prefectures regulate the market entry of institutional services in each region, thus preventing private for-profit providers from entering the market freely, the principle of market competition may not work as intended, and institutional service providers may have less incentive to increase efficiency. With regard to charter status, most studies in U.S. context show that for-profit firms run nursing homes more efficiently than nonprofit or government-owned ones (Nyman & Bricker, 1989; Nyman et al., 1990; Fizel & Nunnikhoven, 1992; Chattopadhyay & Heffley, 1994; Rosko et al., 1995; Ozcan et al. 1998; Anderson et al., 2003), except for those with no statistically significant effect (Sexton et al., 1989; Fizel & Nunnikhoven, 1993; Zhang et al, 2008). However, a more detailed examination is necessary, because there are numerous viewpoints regarding the effect of ownership in the healthcare sector (Herr, 2008; Schwierz, 2011; Tiemann & Schreyögg, 2012); for example, in Germany context, Schwierz (2011) shows that the privatization of the hospital sector may slow down the reduction of excess capacities and be therefore socially wasteful. Furthermore, as pointed out by Tamiya et al. (2011), two-fifths of those certified would not have been eligible if German enrolment criteria had been applied. Therefore, we can say that, thus far, Japan has been over reliant on institutions that are not only expensive but that also endanger the elderly's dignity while offering them poor quality of life. Institutional services have significant potential to increase their efficiency, especially labor efficiency, by reducing services for those needing only minimal nursing care, focusing instead on providing services for those requiring more intensive nursing care.

Another policy implication for efficiency improvements that can be drawn from this study concerns promoting a shift to in-home services, as they can help reduce expenses incurred by Japan's long-term care system. In addition, beyond efficiency considerations, in-home and community-based services can contribute to the goal of the LTCI system, namely, encouraging individuals certified eligible for LTCI benefits to live independently at home as long as possible (Tomita et al., 2010).

Promotion of in-home services requires improving round-the-clock care and respite care. In addition, such a shift requires fostering superior human resources. For instance, Kuwahara et al. (2013) focus on a slightly different topic and conduct a DEA on visiting nurse (VN) service agencies in Japan. They find that relatively efficient VN agencies filled at least 30% of their staff positions with experienced workers.

However, although none of the prefectures show efficiency gains from increasing the number of professional caregivers for institutional services, quite a few prefectures would gain allocative efficiency by increasing capital inputs for institutional services. As this means that preferred policies for promoting efficiency might vary from region to region, scrupulous attention must be paid while drawing possible policy implications from these results. Despite the useful insights obtained, this study has a limitation. To conduct an improved empirical analysis of long-term care performance, we need comprehensive data, especially on operating costs and quality of care (Laine et al., 2005). This goal can be pursued in a future study.

## References

[ref1] Aboagye E, Agyemang O. S, Tjerbo T (2014). Elderly demand for family-based care and support: Evidence from a social intervention strategy. Global Journal of Health Science.

[ref2] Anderson R, Weeks H, Hobbs B, Webb J (2003). Nursing home quality, chain affiliation, profit status and performance. Journal of Real Estate Research.

[ref3] Banker R. D, Charnes A, Cooper W. W (1984). Some models for estimating technical and scale inefficiencies in data envelopment analysis. Management Science.

[ref4] Borge L, Haraldsvik M (2009). Efficiency potential and determinants of efficiency: An analysis of the care for the elderly sector in Norway. International Tax and Public Finance.

[ref5] Campbell J. C, Ikegami N (2000). Long-term care insurance comes to Japan. Health Affairs.

[ref6] Charnes A, Cooper W. W, Rhodes E, Care Work Foundation *Kaigo-roudou no genjyou [Current status of care workers]* (1978). Measuring the Efficiency of Decision Making Units. European Journal of Operational Research.

[ref7] Chattopadhyay S, Heffley D (1994). Are for-profit nursing homes more efficient? Data envelopment analysis with a case-mix constraint. Eastern Economic Journal.

[ref8] Cooper W. W, Seiford L. M, Tone K (2007). Data envelopment analysis: A comprehensive text with models, applications, references and DEA-solver software.

[ref9] Crivelli L, Filippini M, Lunati D (2002). Regulation, ownership and efficiency in the Swiss nursing home industry. International Journal of Health Care Finance and Economics.

[ref10] Erlandsen E, Førsund F. R (2002). Efficiency in the provision of municipal nursing- and home-care services: The Norwegian experience. Efficiency in the Public Sector.

[ref11] Farrell M. J (1957). The measurement of productive efficiency. Journal of the Royal Statistical Society. Series A (General).

[ref12] Fizel J. L, Nunnikhoven T. S (1992). Technical efficiency of for-profit and non-profit nursing homes. Managerial and Decision Economics.

[ref13] Fizel J. L, Nunnikhoven T. S (1993). The efficiency of nursing home chains. Applied Economics.

[ref14] Garavaglia G, Lettieri E, Agasisti T, Lopez S (2011). Efficiency and quality of care in nursing homes: An Italian case study. Health Care Management Science.

[ref15] Herr A (2008). Cost and technical efficiency of German hospitals: does ownership matter?. Health Economics.

[ref16] Hollingsworth B (2008). The measurement of efficiency and productivity of health care delivery. Health Economics.

[ref17] Hougaard J. L, Kronborg D, Overgård C (2004). Improvement potential in Danish elderly care. Health Care Management Science.

[ref18] Ikegami N (1997). Public long-term care insurance in Japan. The Journal of the American Medical Association.

[ref19] Ikegami N, Yamauchi K, Yamada Y (2003). The long term care insurance law in Japan: Impact on institutional care facilities. International Journal of Geriatric Psychiatry.

[ref20] Kawase A, Nakazawa K (2009). Long-term care insurance facilities and interregional migration of the elderly in Japan. Economics Bulletin.

[ref21] Kooreman P (1994). Nursing home care in the Netherlands: a nonparametric efficiency analysis. Journal of Health Economics.

[ref22] Kuwahara Y, Nagata S, Taguchi A, Naruse T, Kawaguchi H, Murashima S (2013). Measuring the efficiencies of visiting nurse service agencies using data envelopment analysis. Health Care Management Science.

[ref23] Laine J, Finne-Soveri U. H, Björkgren M, Linna M, Noro A, Häkkinen U (2005). The association between quality of care and technical efficiency in long-term care. International Journal of Quality in Health Care.

[ref24] Nyman J. A, Bricker D. L (1989). Profit incentives and technical efficiency in the production of nursing home care. The Review of Economics and Statistics.

[ref25] Nyman J. A, Bricker D. L, David L (1990). Technical efficiency in nursing homes. Medical Care.

[ref26] Organization for Economic Cooperation and Development (OECD) Staff (2013). Health at a Glance 2013: OECD Indicators. OECD.

[ref27] Ozcan Y. A, Wogen S. E, Mau L. W (1998). Efficiency evaluation of skilled nursing facilities. Journal of Medical Systems.

[ref28] Rosko M. D, Chilingerian J. A, Zinn J. S, Aaronson W. E (1995). The effects of ownership, operating environment, and strategic choices on nursing home efficiency. Medical Care.

[ref29] Schwierz C (2011). Expansion in markets with decreasing demand-for-profits in the German hospital industry. Health Economics.

[ref30] Sexton T. R, Leiken A. M, Sleeper S, Coburn A. F (1989). The impact of prospective reimbursement on nursing home efficiency. Medical Care.

[ref31] Tamiya N, Noguchi H, Nishi A, Reich M. R, Ikegami N, Hashimoto H, Shibuya K, Kawachi I, Campbell J. C (2011). Population ageing and wellbeing: lessons from Japan's long-term care insurance policy. The Lancet.

[ref32] Thanassoulis E, Silva Portela M. C. A, Graveney M (2012). Estimating the scope for savings in referrals and drug prescription costs in the general practice units of a UK primary care trust. European Journal of Operational Research.

[ref33] Thanassoulis E, Silva Portela M. C. A, Graveney M (2014). Using DEA to estimate potential savings at GP units at medical specialty level. Socio-Economic Planning Sciences.

[ref34] Tiemann O, Schreyögg J (2012). Changes in hospital efficiency after privatization. Health Care Management Science.

[ref35] Tomita N, Yoshimura K, Ikegami N (2010). Impact of home and community-based services on hospitalisation and institutionalisation among individuals eligible for long-term care insurance in Japan. BMC Health Services Research.

[ref36] Tone K (2002). A strange case of the cost and allocative efficiencies in DEA. Journal of the Operational Research Society.

[ref37] Tone K, Tsutsui M (2007). Decomposition of cost efficiency and its application to Japanese-US electric utility comparisons. Socio-Economic Planning Sciences.

[ref38] Tsutsui T, Muramatsu N (2005). Care-needs certification in the long-term care insurance system of Japan. Journal of the American Geriatrics Society.

[ref39] Wong Y. H. B, Beasley J. E (1990). Restricting weight flexibility in data envelopment analysis. Journal of the Operational Research Society.

[ref40] Zhang N. J, Unruh L, Wan T. T (2008). Has the Medicare prospective payment system led to increased nursing home efficiency?. Health Services Research.

